# Smart cities: reviewing the debate about their ethical implications

**DOI:** 10.1007/s00146-022-01558-0

**Published:** 2022-09-30

**Authors:** Marta Ziosi, Benjamin Hewitt, Prathm Juneja, Mariarosaria Taddeo, Luciano Floridi

**Affiliations:** 1 Oxford Internet Institute, University of Oxford, 1 St Giles’, Oxford, OX1 3JS UK; 2 Alan Turing Institute, British Library, 96 Euston Rd., London, NW1 2DB UK; 3 Department of Legal Studies, University of Bologna, Via Zamboni, 27, 40126 Bologna, Italy

**Keywords:** Artificial intelligence, Data privacy, Ethics, Smart cities, Surveillance

## Abstract

This paper considers a host of definitions and labels attached to the concept of smart cities to identify four dimensions that ground a review of ethical concerns emerging from the current debate. These are: (1) network infrastructure, with the corresponding concerns of control, surveillance, and data privacy and ownership; (2) post-political governance, embodied in the tensions between public and private decision-making and cities as post-political entities; (3) social inclusion, expressed in the aspects of citizen participation and inclusion, and inequality and discrimination; and (4) sustainability, with a specific focus on the environment as an element to protect but also as a strategic element for the future. Given the persisting disagreements around the definition of a smart city, the article identifies in these four dimensions a more stable reference framework within which ethical concerns can be clustered and discussed. Identifying these dimensions makes possible a review of the ethical implications of smart cities that is transversal to their different types and resilient towards the unsettled debate over their definition.

## Introduction

Most of the world’s population lives in cities. Cities are the sites where most consumption and production occur and where most of the negative environmental externalities originate (Allam and Dhunny 2019; Yun et al. 2016). In terms of numbers, around 55% of the world’s population resides in cities (Chen et al. 2020), with this figure reaching a peak of 85% in countries like Australia, the UK, and the Netherlands (Metaxiotis et al. 2010). This is why cities rather than nations have become the unit of interest of a substantial part of social, economic and sustainability policy (Praharaj et al. 2018; Yigitcanlar and Dur 2013). This shift has given rise to the idea of using technological innovations to address major urban and societal challenges (Trencher 2019).

Smart cities use technologies like AI and big data for various applications ranging from transportation, trash collection, street repairs, administrative efficiency, surveillance, and more (Kitchin 2018; Sourbati and Behrendt 2021). They can represent a solution to traditional cities’ problems (Csukás and Szabo 2021; Hassan and Awad 2018; Lam and Ma 2019; Zou 2019) as well as entirely new opportunities (Yigitcanlar et al. 2020a, b). While both stances are compatible with a rhetoric of techno-solutionism (Morozov 2013), they entail different framings of a smart city. As solutions to traditional cities’ problems, researchers understand smart cities in terms of their potential to improve efficiency compared to traditional cities. Here, “smartness” can be understood in terms of efficiency gains, where new technologies’ value is defined by their capacity to address the shortcomings of existing approaches to traditional cities challenges. An example would be gathering and analysing traffic data to optimize transport in the city, reduce pollution, and avoid bottlenecks.

At the same time, smart cities bring about entirely new opportunities (Yigitcanlar et al. 2020a, b). For example, the technologies involved in a smart city do not just make the trains run more efficiently, they also enable city officials to collect information about the train schedule and train passengers with techniques including facial recognition scans, gait recognition, body temperature, and more. As the example shows and as we shall discuss in the following pages, this means that smart cities may also include technology and sensors that follow people into their private spaces. Thus, smart cities present both solutions to old problems and new opportunities for the present, and come with their own risks and challenges, which require ethical scrutiny.

The very idea of a smart city is controversial. Its prevalent conceptualisation merely in terms of technology and optimisation potential (e.g. Anand 2021; Yigitcanlar et al. 2020a, b) might eclipse other relevant aspects. Thus, some authors urge acknowledging the complex character of urban life, instead of conceiving the city as an element to optimise (Green 2020; Kourtit and Nijkamp 2012). Inevitably, the definition of a smart city plays an important part in setting the stage for a review of the debate about smart cities’ ethical implications. Concurrently, given that the definition of what may count as a smart city is still contested (Albino et al. 2015; Praharaj and Han 2019), any review that privileges only a specific conception of smart city would struggle to be sufficiently inclusive if not universal in the first place. To bypass the problem, this paper will first provide an overview of the various definitions and labels attached to the concept of the smart city, to then identify four emerging dimensions that are sufficiently common and invariant among the different interpretations. These dimensions will then be used to ground the review. They are the framework within which ethical concerns can be clustered and reviewed. As an analysis of the debate about ethical implications of smart cities, this article does not aim to prescribe a specific ethical framework, but rather identify and analyse existing ethical concerns in the smart cities’ literature. With this framework, we hope to allow future work on ethical concerns to translate across differing definitions of a smart city.

Following this approach, the article is structured in seven more sections. In Sects. 2 and 3, we argue that, even though the definition of smart city is disputed, four dimensions are transversal to multiple definitions (Albino et al. 2015; Yigitcanlar et al. 2020a, b). Then, in the following sections, for each dimension, we present a series of elements of existing ethical concern, identified by conducting a review of the relevant literature. In Sect. 4, we focus on *network infrastructure*, with the corresponding concerns of control, surveillance, and data privacy and ownership. In Sect. 5, we analyse *post-political governance*, embodied in the tensions between public and private decision-making and cities as post-political entities. In Sect. 6, we turn to *social inclusion*, expressed in citizen participation and inclusion, and inequality and discrimination. In Sect. 7, we discuss *sustainability*, focusing on the environment as an element to protect and as a strategic ingredient for the future. In the last section, we draw some general conclusions.

## What is meant by a “smart city”?

The term “smart city” may refer to technological additions to existing cities, or entirely new cities built with “smartness” in mind. The first example of a smart city that comes to mind might be prototypical, either from a Silicon Valley or a Utopian framing (Gibbs et al. 2013; Hollands 2008; March 2016). “Smart city” may refer to citywide efforts to implement new Information and Communication Technologies (ICT) or transportation systems, like in New York or Los Angeles. Alternatively, smart cities may be brand new, entirely constructed cities, like Songdo International Business District in South Korea or the New Clark City (NCC) development project in the Philippines. The expression “smart city” might also refer to the development of a particular neighbourhood, such as Quayside in Toronto or Speirs Locks in Glasgow.

Analysing the host of labels and definitions revolving around the term “smart city” has normative relevance in itself, as it can shed light on the complex dynamics at play. To understand why and how, consider the following three points.

First, the terminological debate is likely to reveal the set of conflicting interests behind the term. The label “smart city” belongs to contemporary jargon around urban development and management. This specialised language is used by consultants and marketing professionals, among others, and it frames how cities are conceived and planned (Praharaj and Han 2019). In the literature, this is expressed by the *citizen-led*, *private-led*, or *city-led* smart city jargon (Cohen and Cohen 2015), as well as in the tension behind *technology-driven* and *human-driven* conceptions of smart city (Echebarria et al. 2020; Kummitha and Crutzen 2017). The technology-driven (also labelled sometimes techno-centric or techno-optimistic) perspective is often found in smart city initiatives spearheaded by US tech companies like IBM (Batty et al. 2012; Kitchin 2014). It focuses primarily on “hard infrastructure”, like ICT, and it usually comes with the assumption that technology has the answer to solve the old challenges that cities face (e.g. traffic). A more human-driven approach is reflected in several European cities, of which Barcelona is an example (Tieman 2017). This approach focuses primarily on “soft infrastructure”, like human and social capital, e.g. education and knowledge (Caragliu et al. 2011; de Wijs et al. 2016; Martin et al. 2018; McFarlane and Söderström 2017). Its perspective is that technology alone is insufficient to meet the challenges of cities, especially without essential lifestyle changes and public policies to preserve and restore urban ecosystems in danger. As these approaches suggest, each label stresses a different connotation which may show or hide the agenda of different actors.

Second, the ambiguity and disagreement around the term “smart city” may also be evidence of a lack of sound theorising behind it (Praharaj and Han 2019). In this respect, many labels indicate the historical process of evolution of the term and its trends. This is expressed by terms such as “*digital city*” (Yovanof and Hazapis 2009), “*tech city*” (Foord 2013), “*wired city*” (Batty et al. 2012), “*ubiquitous city*” (Anthopoulos and Fitsilis 2010), “*intelligent city*”, “*information city*” (Sairamesh et al. 2004), “*knowledge city*” (Yigitcanlar et al. 2008), and “*sustainable city*” (Praharaj and Han 2019). For example, some authors present the relationship between digital, intelligent, and smart in a historical key (Mora et al. 2017). “Digital city” originates from the internet wave at the beginning of the 2000s (Cocchia 2014). And “intelligent city” comes from the meeting of the digital city with the idea of the knowledge society, and it refers to the possibility to use ICT towards human learning and technological innovation (Albino et al. 2015). As for what differentiates the “intelligent city” from the “smart city”, the latter stresses the importance of the institutional and social apparatuses to support policies aimed at forming integrated solutions for different types of city challenges (Ojo et al. 2016). Referring to the term “intelligent”, other authors suggest that a smart city is more user-friendly and accessible than an intelligent city (Albino et al. 2015; Nam and Pardo 2011). Zheng et al. (2020) conceive an intelligent city as the first generation in the wave of urban innovation and consider smart cities as the second generation because of the higher level of participation from urban authorities in the deployment of smart technologies.

Finally, the fact that there is not one single, agreed-upon definition of smart city might be because the concept reflects different perspectives depending on where one is in the world (Praharaj and Han 2019). Not only does it take on different meanings for different people or at different times, but it also means different things in different places. The understanding of “smart city” varies depending on where you are in the world. It changes according to the resources available for innovation, the readiness for change, and the aspirations and expectations of citizens (Praharaj and Han 2019). We have already seen that even North America and Europe differ in their conception of smart cities. The former tends to adopt a technology-driven perspective influenced by the presence of ICT companies such as IBM and Cisco. The latter reflects a leaning towards a low-carbon economy, expressed in the aspirations of the European Union (Mora et al. 2019).

The history and the geography behind the different labelling approaches just discussed bring to light otherwise hidden tensions and divergences and so play an informative role in a normative review of smart cities. However, an excessive focus on the concept rather than on the components of a smart city may take attention away from, for example, the potentially detrimental effects of ICTs on the city environment (Caragliu et al. 2011; Lam and Ma 2019). Additionally, and importantly for this article, the elusive dynamics of labels and concepts might undermine, rather than ground, any evaluation that aims to focus on the overarching, ethical aspects of smart cities, and thus start from an agreed definition. For this reason, the next step is to offer a sufficiently stable understanding of smart cities and their constant features, so that an ethical review of the concerns that smart cities may raise becomes reasonably feasible. This is the task of the next section.

## Re-dimensioning the smart city definition

Even though different conceptions of a smart city mirror different interests, historical trends, and places, some authors have tried to identify a set of dimensions that hold constant across them. In this section, we identify these dimensions in the literature on conceptual frameworks for smart cities, as they provide an analytical clarification of some definitional ambiguities.

A widely adopted conceptual framework (Zheng et al. 2020) maps the concept of smart city on the six dimensions of smart economy, smart governance, smart living, smart people, smart environment, and smart mobility (European Parliament 2014; Giffinger et al. 2007). While this framework is sufficiently broad to cover a variety of smart-city projects (Cocchia 2014), its “smart” labelling might encounter the same problems against which we have warned in the previous section. For it leaves open what makes a city, as much as an economy or a kind of governance, “smart” in the first place.

In an attempt to circumvent such circularity and the ambiguity concerning the smart city label, Yigitcanlar et al. (2018) created a “global” conceptual framework to examine smart cities practices across the world. By reviewing 78 definitions of smart city, they identified (a) economy, in terms of productivity and innovation; (b) society, in terms of liveability and wellbeing; (c) governance, in terms of governance and planning; and (d) environment, in terms of sustainability and accessibility as the main smart city development dimensions.

Although these dimensions are sufficiently general, they still leave out a crucial aspect of smart cities: technology. Several authors warn against characterising smart cities merely in relation to technology (Glasmeier and Christopherson 2015). However, its role as an essential component in defining a city as “smart” cannot be denied nor omitted. In this respect, Caragliu et al. (2011) identified aspects common across smart cities by devising a framework meant to unpack what makes a city “smart”. These are “(1) the use of networked infrastructure to improve economic and political efficiency and enable social, cultural and urban development, (2) an underlying emphasis on business-led urban development, (3) a strong focus on the aim of achieving the social inclusion of various urban residents in public services, (4) a stress on the crucial role of high-tech and creative industries in long-run urban growth; (5) a profound attention to the role of social and relational capital in urban development, and (6) social and environmental sustainability as a major strategic component for smart cities” (Caragliu et al. 2011, p. 67).

On a similar note, by reviewing more than a dozen definitions of smart cities, Albino et al. (2015) isolated four prevailing aspects that enable one to identify a city as “smart”. These are “(1) the presence of a city’s networked infrastructure that enables political efficiency and social and cultural development, (2) an emphasis on business-led urban development and creative activities for the promotion of urban growth, (3) social inclusion of various urban residents and social capital in urban development, and (4) the natural environment as a strategic component for the future” (Albino et al. 2015, p. 13). Both approaches unpack, rather than rely on, the term “smart”. Additionally, they are general and yet specifically tailored around the characteristics of a smart, rather than any other traditional city.

Taking inspiration from the above studies, and by merging the last two frameworks, in this article we identify (a) network infrastructure, (b) post-political governance, (c) social inclusion, and (d) sustainability as the four dimensions of the conceptual framework that best accommodates the review of a series of ethical concerns identified by a systematic search (Grant and Booth 2009) of the *relevant* literature on smart cities. “Relevant” here qualifies articles that appeared in a systematic search across four databases,^1^ for the main terms of (“smart city” AND ethics), iteratively complemented by a theme- or topic-specific search for each of the four identified dimensions of our framework. The search process for each dimension was guided by theme-specific words, such as “connectivity” and “transportation” for (a) Network Infrastructure, or “environment” for (d) Sustainability (see full “Methodology” in Appendix).

As indicated in the introduction, smart cities can represent the solution to traditional cities’ problems (Csukás and Szabo 2021) and be catalysts of new opportunities (Yigitcanlar et al. 2020a, b). In this respect, a review on the ethical aspects of smart cities should consider that some of the challenges it identifies might simply be longstanding issues relating to urban development, rather than novel problems. Indeed, some common concerns about smart cities, from increased surveillance to accessibility of services, may be the same issues affecting traditional cities. At the same time, such a review should identify new ethical concerns, which may not be found in a more traditional conception of the city, but rather arise from affordances unique to a smart city. To draw this distinction clearly, the rest of the article will present each dimension in relation to its present role in a smart city and its past in a more traditional city context. This strategy will help to structure the review along a narrative that sheds light on the ethical concerns raised by smart cities as solutions to traditional cities’ problems and ethical concerns related to the new opportunities, unique to smart cities.

## Network infrastructure

Some argue that the smart city wave we are seeing now is only the most recent development of a longstanding trend. Pointing to bureaucratic modernisations and new knowledge technologies of the nineteenth century, Robertson and Travaglia argue that we are in the midst of a second big data revolution that differs in volume and velocity of data but presents many of the same challenges (2015). While one can admit that techniques, such as data collection, often target now, as then, groups marked as “moral outsiders” (Robertson and Travaglia 2015), one should also note that the data collection involved in contemporary smart cities occurs with unprecedented granularity and seamless efficiency, affecting citizens’ lives and relationships with government and the city in profound and rather unprecedented ways (Yigitcanlar et al. 2020a, b).

We find ourselves in an information age, generating and relying on data like never before. It is a new stage in human evolution (hyperhistory), wherein information and communication technologies record, transmit and process data, with human societies crucially relying on ICTs and on information as an essential resource (Floridi 2012). In this new stage, concerns around a city infrastructure are not solely about urban planning, but they extend to the whole network of technologies that pervade the smart city. These technologies may include, among others, big data analytics, cloud computing, IoT, blockchain, robotics, 3D printing, 5G and Artificial Intelligence (AI) (Yigitcanlar et al. 2020a, b). Here, we identify the ethical concerns relating to this “networked” infrastructure as issues of *control*, *surveillance*, *data privacy* and *security*. As we will show, these points are highly interconnected. For example, a simple outage suffered by a private company such as Facebook in October 2021 (Talmazan 2021) can lead to loss of control over government services (e.g. it caused a disruption of healthcare, education, and other government services in cities across the globe), potential loss of essential and private data as well as lead to an increase in surveillance methods following the incident in the name of improved security.

### Control

The centralisation of data in smart cities gives considerable power to those who control it. There are different kinds of control in play here. One is the control of architecture, on what can be done physically within the boundaries of a space. This kind of control in cities is not new and falls under the category of urban planning typically discussed in traditional cities. The other kind of control is that of data and knowledge. This refers to a network rather than an urban infrastructure.

As cities become “smarter” and increasingly connected with sensors and reliant on algorithms being fed large quantities of real-time data, the power centred in the administration of city services moves from the mayor’s office and city council chambers to the control rooms, from officials who are responsive to democratic will to those processing the data. Control rooms in cities are not new. However, as Kitchin notes (2015), they are becoming more consolidated and streamlined. Early control rooms were siloed and dealt with monitoring and managing closed systems like an electricity grid. Now, control rooms are not only broader in their remit, but they are also increasingly automated, sometimes with humans-in-the-loop who can actively intervene, enacting what Dodge and Kitchin call “automated management” (Dodge and Kitchin 2007). As an example, Greenfield (2013) and Kitchin (2015) point to the Intelligent Operations Centre in Rio de Janeiro. Built by IBM, this $14 million facility brings together in one place real-time data from thirty different agencies. This includes data from traffic cameras, social media posts, weather stations and police patrols.

Although smart city technologies can increase the control of the government over people, they can also shift that control to private entities. Fisher (2020) brings the notable example of Waze, a navigational app offered directly to consumers, unlike most smart city technologies, although Waze functions in much the same way as many smart city projects. Waze collects real-time data from millions of drivers’ devices to deliver a personalised service, in this case directions to best navigate traffic. In doing so, it redirects traffic through side streets and residential neighbourhoods, causing tension among residents over the management of traffic, traditionally in the public sector’s control.

The degree of control afforded to officials in smart cities exceeds that of any previous era. Those in, or otherwise responsible for, control rooms can monitor and affect city activities and systems with extraordinary detail and pervasiveness. Such power can be used well, but also misused or even underused (opportunity costs and ethically wrong omissions), and in each case there is a pertinent moral dimension to be considered and addressed, possibly leading to regulations.

### Surveillance

A major ethical implication of smart cities concerns the surveillance of their citizens. Surveillance as a consideration for urban planning is not new. In a related sense, for example, Georges-Eugene Haussmann, the architect of the great boulevards of Paris, acknowledged the military value of having broader and straighter streets to dispatch riots quickly, allegedly using this justification to attain more funding for his projects (Andrews 2017). The same issue seems to be reappearing in the restructuring of El Cairo (Lewis and Ebrahim 2020). However, this is an area where the deployment of ICTs around the city departs from the past, especially since the onset of COVID-19. For instance, it is estimated that there are around 691,000 CCTV cameras in London (CCTV.co.uk 2020). Israel is using a facial recognition surveillance system that can detect individuals through face masks (Halon 2020). And even before the novel Coronavirus, the smartphones that many people carry around in their pocket relayed detailed location tracking information (Thompson and Warzel 2019).

Yigitcanlar et al. (2020a, b) report that state-of-the-art AI surveillance technologies can be applied for the monitoring of communication networks, and to recognise threats, from accidents and fire to crime and fraud. These technologies include predictive analytics, drones, motion detection and other autonomous devices. All this can help cities improve their services and economic and security status (Allam 2019). In this respect, surveillance is often closely associated with the optimisation of services (e.g. urban services) and, more often, with an increase in security and prevention. However, it can also, and very easily, be used to control and influence citizens’ behaviour in extraordinary detail and pervasiveness. For example, ‘smart streetlight’ cameras in San Diego were initially introduced to help city officials study traffic patterns, but were later regularly used by police officers to investigate purported crimes (Holder 2020).

In cases like this, smart cities may run the risk of becoming a tool or even a catalyst for unwarranted surveillance as well as exacerbating existing inequities in policing systems in the name of increased security. Additionally, some smart cities may install surveillance tools specifically for policing, raising additional ethical questions. In the city of Chicago, “Shot Spotter” gun-shot-detection boxes placed at streetlights around the city are meant to use AI to detect the sound of gunshots to prevent crimes from going unreported and speed-up responses. Work has since shown that Shot Spotter is unlikely to have a significant impact on firearm-related homicides or arrests (Doucette et al. 2021), while there have been cases of people wrongfully jailed because of the technology (Burke et al. 2021). Other surveillance-related smart city technologies, such as “predictive policing” programs that seek to help optimise routes for police officers, may suffer from data biases, sending police to areas with high crime rates simply because they have historically been policed more often (Ferguson 2016).

Peculiar to smart cities is the fact that people themselves are also participating in their own surveillance, including through wearable devices. Clarke and Steele (2011) argue that personal fitness tracking devices and data can be used in smart cities to inform public health and population health data, urban planning and environmental monitoring, fitness trends and social network analysis, and personalisation of health information. Other scholars have found that such self-quantification has ambivalent or even conflicting effects, being empowering, disempowering, and overpowering (Mau 2019). The emergence of self-quantifying devices has led to what De Moya and Pallud (2020) call the heautopticon, a panopticon applied on oneself by oneself. Additionally, Manokha argues that employers are increasingly turning to surveillance measures of this kind to control their workers and increase productivity (Manokha 2019). Regardless of the merits of Manokha’s specific claim, it is hard to dispute that cities and employers are both, and sometimes in tandem, increasing surveillance measures in the name of efficiency. This raises ethical concerns over individual autonomy as well as privacy.

### Privacy and security

In line with what has been presented above, new technologies enable multiple stakeholders and government bodies to collect real-time data, analyse it and to act quickly in response. This may promote an increased level of security and of protection of privacy compared to traditional cities (Allam 2019). At the same time, the pervasive deployment of ICTs makes cities vulnerable to data security problems, such as data breaches or cyberattacks (Lam and Ma 2019). Additionally, their pervasive process of data collection presents a challenge to data privacy (Pavlou 2011; Price et al. 2005).

On the one hand, a smart city can present privacy and security as improvements over a traditional city’s problems. In terms of privacy, van Zoonen (2016) notes that a great portion of the data used in smart cities is impersonal data, often used to improve a city’s services. This highlights an arguably more beneficial use of data rather than surveillance, and the use of impersonal over personal data. In terms of security, some authors speak in terms of a “safe city” (Allam 2019) when referring to security in relation to smart cities. In particular, Lacinák and Ristvej (2017) report that the concept of “safe city” generally refers to increased security in cities in terms of tackling urban conflicts and crimes, as well as to violence prevention in the context of urban tensions such as forced evictions, land conflicts and scarce urban resources. Additionally, Edwards (2016) argues that both privacy and security are important to smart cities as a prerequisite to keeping the trust and engagement of smart city residents.

On the other hand, authors like Hassan and Awad (2018) and Lam and Ma (2019) report concerns around privacy and security that derive specifically from smart cities’ use of new technologies and their increased level of connectivity. In terms of privacy, Ziegeldorf et al. (2013) identify seven privacy threats specific to IoT use in Smart Cities. These are: “identification, localization and tracking, profiling, privacy-violating interaction and presentation, lifecycle transitions, inventory attack and finally, linkage” (Ziegeldorf et al. 2013, p. 2734). User profiling is considered as a major threat among them. Additionally, Caron et al. (2016) highlight how the use of increasingly complex technologies in a smart city allows a great amount of data about citizens to be collected. This often happens without them being asked for consent nor being given an explanation about why the data is collected and how it will be used.

In terms of security, Yigitcanlar et al. (2019) call attention to how the reliance on cyberinfrastructure, often considered the core fabric of smart cities, makes them vulnerable to cyberattacks. Data centres might be hacked, and data can be stolen or intercepted in transit (Mohamed et al. 2020). Besides cyberattacks, Lam and Ma (2019) stress errors in design, the complexity of large and interdependent systems involving multiple stakeholders, and weak encryption as other major causes of security breaches. The effects of security breaches can be highly damaging both for the city and the individual citizens. Examples vary from loss of control over and the potential failure of a city’s systems and non-availability of essential services (McClure et al. 2001) to breaching the confidentiality of citizens’ data (Ferraz and Ferraz 2014) and losses at the economic level (Mok 2014; Yadron 2016). In 2021, an Amazon Web Services outage caused significant disruptions to internet traffic, including an hour-long outage to the UK government’s “gov.uk” website (Hern 2021). Given the often cloud-focused implementation of ICTs in smart cities, it is not hard to imagine vital government services going offline for significant periods of time. For example, the October 2021 Facebook outage may have disrupted healthcare, education, and other government services in cities across the globe (Talmazan 2021).

While security and privacy are a prerequisite for citizens’ trust and engagement, their failure and abuse can risk public trust and threaten democracy. Techniques such as security and privacy (SPE) enhancement framework can support potential mitigation strategies (Krupp et al. 2017). Nevertheless, these represent technical fixes with social implications and impacts not yet fully explored (Hassan and Awad 2018).

## Post-political governance

The “post-political” can be understood as a reliance on market mechanisms and privatisation with the added backing of technology to appear objective (Beveridge and Koch 2017). The appeal of easy and efficient solutions that appear to be objective raise the question of whether smart cities may represent a new model of governance, called post-politics. In this section, we analyse how the transforming effects of smart cities challenge the traditional roles of, and boundaries between, public and private decision-making, and the conception of the city itself, seen as a post-political entity.

### Public and private decision-making

The previous paragraphs mostly depicted the dangers of excessive government power and control, but smart cities are also places that reveal government dependency on private actors. For example, when cities contract with private entities to transform aspects of their public services, or when national governments do so to build new smart cities from scratch, they cede some degree of decision-making power to the group designing the digital solutions. This shift in decision-making power questions the traditional conception of governance as the monopoly of the government.

In this respect, Meijer and Bolívar (2016) present four configurations for smart city governance: governance of a smart city *tout court*, smart decision making, smart stewardship, and smart urban collaboration. These configurations represent four theoretical perspectives on the role that governance can play in a smart city. And in turn, these perspectives differ in what they envision as the degree of transformation needed in governance to make a city “smarter”. The most conservative conceptualisations describe the preservation of existing institutional agreements towards the creation of smart cities. Instead, more extreme conceptualisations suggest that governance itself should be transformed for a city to achieve a “smart” status. In this respect, the fourth level of conceptualisation is the most transformative, as it envisions smart governance in terms of intelligent collaboration among the multiple actors in the city (Echebarria et al. 2020).

These different configurations of governance may raise questions about the legitimacy of government bodies, as they employ predictive algorithms and data-processing software that they did not produce and may not fully understand. These tools may also present problems for government transparency; while many governments allow residents to send public records request to view government information (e.g. Freedom of Information requests in US and UK), the decision-making processes of black-box predictive algorithms are often un-interpretable to even the developers themselves. Guidelines and internal documentation can explain decisions made by humans, but the use of non-transparent technology in smart cities may complicate public oversight. When private companies develop these technologies for public use, transparency becomes even more problematic.

Calo and Citron (2020) write about the use of automation in US federal agencies, detailing the trend of government agencies to automate their specially delegated power based on expertise and discretion, creating a crisis of legitimacy. They note that nearly half of all federal agencies are using or looking into using AI. At the same time, authors like Yigitcanlar et al. (2020a, b) stress the need for governments and municipalities to assess their digital infrastructure first. On that matter, the Covid-19 pandemic has shed light on governments’ technological inefficiencies, with several cases like the Queensland state government in Australia, which, at first determined to offer education online, saw its infrastructure failing due to excessive web traffic (Yigitcanlar et al. 2020a, b). Additionally, technology professionals claim that just 15–20 per cent of large public sector technology projects are successful, partly because of poor planning and procurement, and partly because of mid-project changes in scope (Susskind 2019).

The lack of readiness of the government to harness innovation provides opportunities for private companies to take over and re-shape the rules of previously public services. Platforms like Airbnb and Uber offer good examples of the case in point. Their increased use impacts urban settings (e.g. changes traffic flows) and the balance of responsibilities between the public and the private sectors [e.g. see Uber Files for examples on Uber’s role in lobbying government and defying the law (Davies et al. 2022)]. Private interests have always shaped urban housing and transportation to some extent, but the reach and tactics of these companies constitute a shift in kind (Davies et al. 2022). Consider for example, the reports of alleged attempts of Uber senior executives to lobby head of states or the accusation against Uber to thwart law enforcement using a “kill switch” to hide data from police during raids (Davies et al. 2022).

The success of Uber epitomises the trend of increasing privatisation and individualisation of public services. Few public services draw as much attention from policymakers and, quite often, from city residents as these platforms. At the same time, Uber also demonstrates the utility of transport data to deliver a more effective service. In Brighton and Hove, for instance, researchers found that community transport systems and the local governments commissioning them did not utilise data in a structured or effective way, compared to Uber’s “data-first approach to transport” (Sourbati and Behrendt 2021). Although they are not the prototypical examples of smart cities, these platforms demonstrate the trend of privatisation and datafication of services formerly guaranteed by public entities. In cities like San Francisco, where government agencies have partnered with companies like Uber, platforms have been integrated into the smart city framework (Khosrowshahi 2018), only further stressing the need for close ethical consideration.

### Cities as post-political entities

The smart city features not only different configurations of governance, but also different modes of governing. Kitchin captures this idea in the shift from data-informed to data-driven urbanism (2015). New technologies such as AI and data analytics can speed-up the decision-making process by analysing a large amount of data to inform decisions. These new technologies can also automate this process of decision-making by letting the results of these complex analyses determine rather than simply inform decisions. While the former aspect can be conceived as an increase in efficiency from past modes of decision-making, the latter presents a new scenario where decision-making becomes entirely (or almost entirely) automated (Dong et al. 2019; Soomro et al. 2019; Yu et al. 2019). This process of increasing automation comes with advantages as well as risks. On the one hand, the International City/County Management Association (ICMA) lists, among the advantages of automation, the possibility for local governments to run more efficiently, to focus on their residents, to tackle human bias, and to optimize the use of public funds (ICMA 2019). On the other hand, some authors stress how increasing automation can pose a threat to social inclusion and participation (Barocas and Selbst 2016), exacerbate existing bias and inequality (O’Neil 2016), and provide austere solution to existing economic and social crises of cities (Gray 2018). Automation can also undermine accountability.

With regards to urban choices more specifically, Neil Gray (2018, p. 1) describes the post-political as a cover for what he calls “soft austerity urbanism”, which he defines as “seemingly progressive, instrumental small-scale urban catalyst initiatives that in reality complement rather than counter punitive hard austerity urbanism”. Masked by the cover of innovative sounding programs often shortened to acronyms, Gray points out that such programming actually served to reduce 10,4000 social rented homes in Glasgow, displacing many local inhabitants. These programs fit the post-political profile, as initiatives guided by a reliance on market mechanisms and privatisation with the added backing of technology to appear objective (Beveridge and Koch 2017). However, Beveridge and Koch (2017) push back against the inevitability of this post-political stage, arguing that depoliticisation is a dynamic and contingent process that can reframe the scope and stakeholders of conflicts, rather than necessarily skirt such conflicts. Davidson and Iveson (2015) describe a framework for conceiving the city as a political entity that moves beyond the rhetoric of post-politics and responds to all constituencies and their grievances. This shows how the post-political idea associated to smart cities can be seen as mimicking old solutions to old problems (e.g. see austerity urbanism example above) or as bringing about new opportunities to depoliticise conflicts between stakeholders in a smart city through data-driven policy decisions.

## Social inclusion

The benefits of smart cities depend on what these cities envision as smart citizens. This determines which benefits (if any) citizens receive and who receives those benefits. In this context, we identify citizen participation and inclusion as well as inequality and discrimination as key points of relevance. Overall, which citizens get to participate in the shaping of a smart city, to what extent and on which issues are key issues in matters of social inclusion.

### Citizen participation and inclusion

The input and participation of urban residents are essential for the fair and effective deployment of smart city technologies. Therefore, some retain that cities can be defined as “smart” only when they successfully integrate the democratic participation of their multiple stakeholders, among which citizens, within a city’s management (Fernandez-Anez et al. 2018). The literature presents different avenues for citizen participation. They vary from innovative projects, such as digital urban platforms, where citizens can vote on urban initiatives but also collaborate and solve specific problems together, to citizens acting as sensors for private or public bodies by, for example, flagging problems or creating content. These modes of participation vary in envisioning a more active or passive role for the citizen (Gooch et al. 2015; March and Ribera-Fumaz 2018; Waal and Dignum 2017). Examples of digital platforms where citizens can vote and come up with proposals for the city are e-democracy platforms such as Decidim.^2^ Examples of initiatives that allow citizens to co-create and engage in collective “problem-solving” are fablabs (Trencher 2019). These, like other ‘makerspaces’, are creative places for people to gather, learn about and employ manufacturing technologies and digital design to make things in collaboration (Hielscher and Smith 2014; Smith et al. 2016).

Nevertheless, several authors are critical of these initiatives. Some argue that digital solutions like e-democracy platforms and fablabs are designed with tech-savvy people in mind, as they require good data literacy levels or programming skills (Trencher 2019). Furthermore, the role of citizens in them is secondary and engaged in problem-solving activities that only have an indirect impact on the city. The role of citizens is often limited to reporting problematic conditions, like potholes, or simply voting on ideas worthy of funding (Cowley et al. 2018). Smith et al. (2016) suggest that these initiatives serve instead to reveal the inability of the current political-economic system to adapt successfully to the need for new forms of production and consumption centred around citizens, democratic ideals, and sustainability. Overall, there is still much work to be done in actively integrating citizens in shaping the city, as much as in adapting the understanding of participation to the new conditions afforded by the smart city. The failure to address this can have major consequences for democracy and may exacerbate inequality and discrimination in the city.

### Inequality and discrimination

The benefits of smart city technologies may not reach all city residents equally, and their deployment may exacerbate longstanding inequalities. Cities are sources of great prosperity and GDP output, but they also feature sharp divides between the rich and the poor and other social, digital divides, often with devastating effects. In Chicago, there is a 30-year gap in life expectancy between rich and poor neighbourhoods (City Health Dashboard 2015). In the San Francisco Bay Area, more than 120,000 workers commute more than 3 h each day because of a lack of affordable housing (Board 2020). There is great inequality in cities today, as in the world generally, and without due consideration, smart cities may accelerate rather than ameliorate this divide.

Many people lack the digital literacy skills, the technologies, or sufficient internet connection to use smart city technologies. According to Pew Research Center, about 75 per cent of Americans in urban areas have broadband at home, which is 12 percentage points higher than those in rural areas (Vogels 2021). Another Pew study found that 14 per cent of U.S. adults have low digital skills and low trust in online information (Horrigan 2016). Similar gaps are part of the digital divide and are likely to impact the effectiveness of smart city technologies. When cities, say, move benefit sign-ups and other crucial forms online, they may create new inequities, or exacerbate the same inequities the transition was meant to solve.

The use of the technologies themselves, regardless of the population’s connectivity, may also entrench inequalities. Much has been written about the problems of fairness in the use of algorithms. City officials and other customers of smart city technologies like to point to the outcomes of algorithmic predictions and decisions as objective and unburdened by value judgments. However, these algorithms are trained with data from the “real world,” which invariably reflects ethical and political choices and historical trends that may be open to criticism. Algorithms that help decide who should get a bank loan, the economic impact of routing a new highway through poor neighbourhoods, or where police should send more patrols, are informed by, and reinforce economic and racial disparities (Yigitcanlar et al. 2020a, b), tending to punish the poor (O’Neil 2016).

The benefits of smart cities depend on what the cities envision as smart citizens, those who are in the position to exploit, or have access to, the means and the knowledge required to use the available resources in the best way (Gran et al. 2021; Janssen et al. 2015). Older people and ethnic minorities, for example, are often left out of data sets and further marginalised by technological innovation (Sourbati and Behrendt 2021). Thus, smart cities must consider the impact of their technological deployments on the goal of data justice, or the fairness in the way people are made visible, or not, in their handling of digital data (Taylor 2017). The smart city often caters primarily to entrepreneurs and high-skill professionals as its “smart citizens”. By attracting these into newly developed neighbourhoods or cities, smart cities can raise home prices and accelerate gentrification. Scholars have noted that smart cities can end up replacing existing populations by tearing down old buildings or neighbourhoods to make room for new developments, which often include insufficiently affordable accommodations (Gray 2018). This is why Shamsuddin and Srinivasan (2021) argue for more attention to the needs of vulnerable groups, specifically relating to housing, to build more inclusive smart cities.

## Sustainability

Sustainability should not be understood one-dimensionally, merely in relation to the environment. Bibri (2020a, b), for example, defines the three dimensions of sustainability as the social, the economic and the environmental. However, as smart city projects are often justified by making references to the environmental dimension (Albino et al. 2015; Yigitcanlar et al. 2020a, b), we will specifically focus on the latter. In this respect, we shall see in this section that the environment is alternately understood as an element to protect and as a strategic component for the future.

As an element to protect, smart cities can help mitigate the adverse effects that traditional cities had and keep on having on the environment. Some authors argue that urbanisation has had profoundly adverse effects on the environment due to excessive urban growth (Dodman 2017; Estevez et al. 2016; Han et al. 2017). These effects include environmental degradation, air and water pollution, resource depletion and intensive energy use, inefficient planning systems and the mismanagement of facilities, poor housing and working conditions, public health and safety hazards, the exacerbation of inequalities and so on (Bibri 2018). New technologies can help to mitigate these effects, for example, by introducing smart energy systems to minimise energy consumption and production, by monitoring and anticipating changes in the environment, or by operationalising more efficient transport systems (Yigitcanlar et al. 2020a, b). For example, the city of South Bend, Indiana, which has a population of just over 100,000 people, saved $437 million by implementing “smart sewers” that optimise the traffic of wastewater (Blasko 2021). Positive about initiatives like these, some authors (Dodgson and Gann 2011; Pham 2014) have emphasized the difference between environmental preservation and economic growth, claiming that smart cities initiatives can contribute to both. However, others are sceptical about the compatibility of these goals (Hollands 2008). Bibri (2020a) argues that the economic dimension wins over the social and environmental one and that smart city projects prioritize the efficiency of solutions rather than providing solutions for sustainability challenges.

Several initiatives show how smart cities can relate to the environment as a strategic element for the future. For example, Bibri and Krogstie (2020) list smart grids, meters and buildings, as well as smart urban metabolism and environmental monitoring as examples of some technological solutions for environmental sustainability in smart cities. They are positive about the capacity of these solutions to produce, all combined, a greater positive environmental impact than the sum of their individual effects. This impact can entail improvements in energy efficiency, a decrease in environmental pollution and a shift towards renewable energy (Bibri and Krogstie 2020). However, they also acknowledge that several of these initiatives come at a great cost for the environment, given the negative impact of the technologies involved. Behind their virtual appearance, there hides a very material side to smart city solutions (Berkhout and Hertin 2004; Williams 2011). Large quantities of scarce elements, like rare earth minerals and critical metals, are required to develop these technologies (Chancerel et al. 2015). Additionally, the extraction of such materials might lead to socio-environmental impacts and conflicts in the territories of interest. Their recycling also represents a significant concern (Ali 2014). Technology use also necessitates energy use, and as smart cities begin to embrace technologies like blockchain—the newly elected mayor of New York City has requested that some of his salary be paid in Bitcoin (Banjo and Maglione 2021)—smart cities may present serious ethical questions about energy consumption (Cowls et al. 2021a, b). The relation between smart city technologies and environmental improvement is not unidirectional, but full of complexity and uncertainty (Berkhout and Hertin 2004). Further research should investigate this intricate relationship. Failing to do so might result in a Trojan horse, where we welcome future disasters as solutions to present problems.

## Conclusion

Both as solutions to traditional city problems and as new opportunities for the present, smart cities come with their own risks and challenges, which call for ethical scrutiny. As solutions to traditional city problems, smart city projects are often conceived as an increase in efficiency over previous approaches. This feeds into a conceptualisation of smart cities mostly in terms of technology and optimisation potential which, as stated in the beginning, might eclipse the complex character of urban life and its multidimensional challenges. When unquestioningly introduced, these technological solutions might incur the danger of exacerbating pre-existing problematic aspects of old solutions as well as generating new ones. As we have seen in the section on surveillance, technologies that entail predictive analytics, motion detection and autonomous or semi-autonomous devices such as drones are often introduced in the name of increased protection and improvement of a city’s traditional services and its social and economic status. However, they can pose a threat to the autonomy and privacy of citizens. Additionally, as shown by the example of San Diego, even when introduced for seemingly beneficial purposes—such as “smart streetlight” cameras to understand traffic patterns—these initiatives can serve controversial purposes, such as increased policing, which can, in turn, exacerbate existing inequities and introduce new forms of bias and discrimination. It is paramount that smart city projects, when presented as new solutions, are informed by the problems that made previous solutions problematic or redundant. Additionally, when conceiving smart cities as solutions to current social, environmental, and economic problems, it is crucial to assess their potential for direct and indirect social, environmental, and economic impact.

Some of these impacts are intuitive, and easily discoverable. It is clear that policies of increased technological surveillance in policing, potential downtime of vital city services because of private company outages and shifts from public accountability to privately led projects deserve consistent ethical scrutiny and rectification. However, smart cities bring other, often inter-linked, potential ethical issues, which may be less visible. Increased surveillance from traffic sensors shifts the responsibility of safety and neighbourhood management from city officials to traffic control rooms. In the name of increased accessibility, the digitisation of government forms may do the opposite and translate inequities in digital access to inequities in government services. The optimisation of city services through technology may result in social service austerity, leaving behind groups whose needs are not easily quantifiable in models. Due to their ease of scale, even small changes in smart city technologies—from the location of sensors to the way buttons are placed on city websites—can significantly impact the lives of residents.

As new opportunities for the present, the information revolution which powers smart cities has led to redesigning the environment surrounding us to make it more digitally friendly (Floridi 2019). These transformations have profound ethical, social, and legal implications (ELSI). For example, when applied to cities, they imply changes in the way citizens access services and can exert their rights. For smart cities to hold their promise to improve individual lives, social wellbeing, and environmental conditions, it is essential to consider the many aspects and implications of these transformations, anticipate or minimise problems, and provide redressing opportunities. These measures affect a large spectrum of features characterising smart cities, from procurement policies and public–private partnerships to policy strategies and unintended side-effects. In many cases, lessons about the ELSI of digital transformation can be learned from domain-specific cases, and work on smart cities should build on this expertise. When considering the urban environment, an extra challenge emerges with respect to how problems may be intertwined. Solving them in a satisfactory way often requires careful balancing of competing interests and rights, and ultimately political strategies able to understand both opportunities and limits of the digital transformation and align them with the values of our societies.

The four dimensions identified here provide the groundwork for an ethical analysis of a fast-changing field that can offer many solutions and opportunities, as well as for tracking the multiple aspects in which smart cities reinforce old, and introduce new, ethical challenges.

## Figures and Tables

**Fig. 1:**
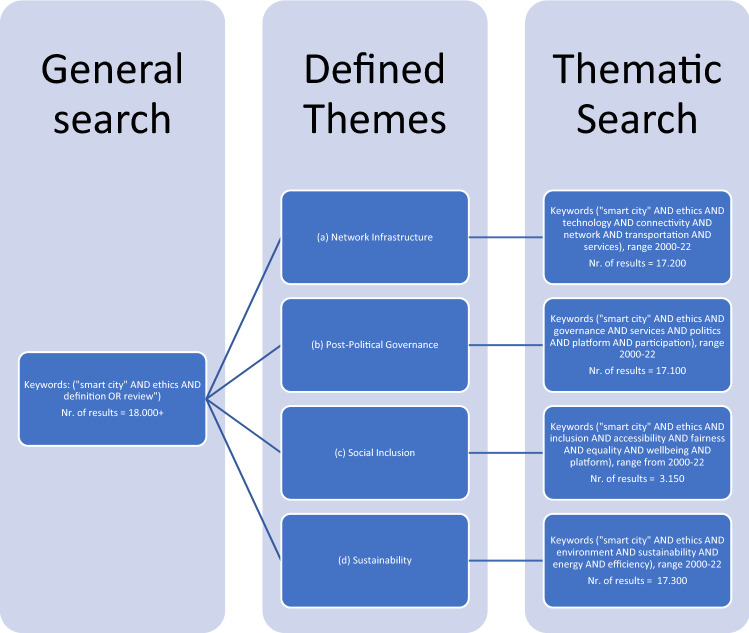
General search results

**Fig. 2:**
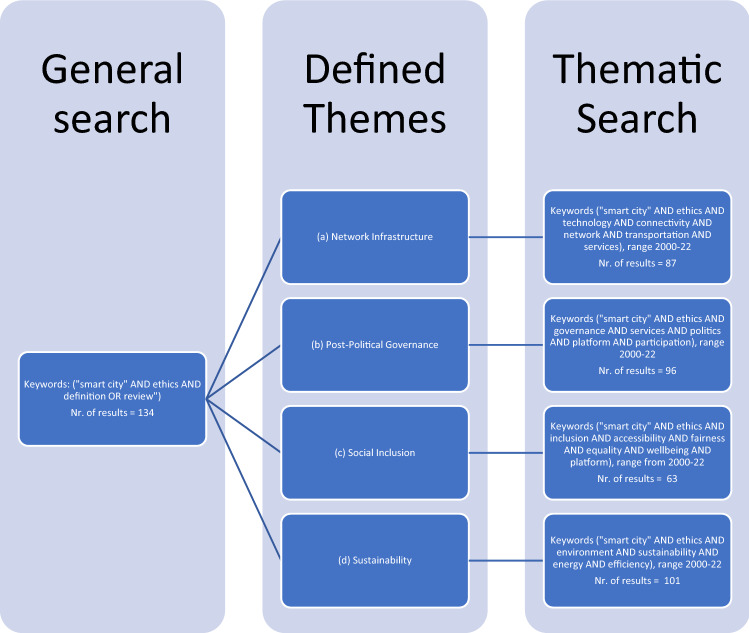
Reviewed search results

## Methodology

This review of the debate about Smart Cities' ethical implications was conducted by means of a systematic search and review of scholarship relating to smart cities and ethics (Grant and Booth 2009). This type of review entails a comprehensive search process, allowing the incorporation of multiple study types (Grant and Booth 2009). This is suitable for this case, where the reviewed scholarship included papers from multiple disciplines (from Ethics to Science and Technology Studies to Urban Studies, etc.), as well as different study types (research articles, metareviews, literature reviews, etc.), These studies were collected primarily from top research databases: Google Scholar, PhilPapers, Scopus, and Web of Science.

The search process was split into a general search and a series of more thematic searches. The relation between the two types of searches is chronological. The general search was used to identify definitions of smart cities and frameworks used to categorize them. We elaborated on them in the corresponding Sects. 2. and 3 of this paper. Once we identified four dimensions of interest from an analysis of smart city definitions and frameworks, we conducted a more targeted thematic search. This informed Sects. 4, 5, 6 and 7 of this paper. Figure 1 shows the keyword search, the selected time range, and the number of results (aggregated across the multiple search engines considered) for each search. As it can be observed from the keywords used, none of the words that head the subsections of each dimension in the paper (e.g. surveillance, privacy and security for the dimension of “Network Infrastructure”) was directly used in the search. These are sub-themes that emerged during the review process, which followed an inductive approach. Additionally, each initial search returned around 17,000 results (apart from the Social Inclusion one, which returned 3150). At this stage, it was paramount to adopt a strategy to reduce this number to a manageable size.

To reduce the number of results, “Publish or Perish”^3^ (PoP), a freely available software program for retrieving and analysing academic citations, was used to re-run the above queries. PoP allows to analyse papers according to a range of citation metrics (e.g. total citations, h-index), year of publication, journal and type of publication (e.g. article or book). On one hand, PoP was used to validate the results of the initial search (only the searches on Google Scholar, Scopus and Web of Science were re-run as PhilPapers is not available on the software). On the other, the software was used for data cleaning and to determine relevance. Data cleaning was conducted by removing duplicates, assessing the overlap between the targeted searches, and by removing papers with equal or less than one citation from our dataset. A screening process for relevance to the ethics of smart cities as well as to the topics of each specific theme was conducted by reading each remaining title and, where in doubt, retrieving the abstract. Specifically, we made sure that the process of syntactic relevance in our search matched that of semantic relevance (e.g. that keyword “wellbeing” was not about specific health apps but about the quality of life in a smart city in general). Overall, this process allowed us to cut down the total number of papers to review to the numbers expressed in Fig. 2. These are the papers that were reviewed.

## Footnotes

1 The specific databases were Google Scholar, PhilPapers, Scopus, and Web of Science.

2 https://decidim.org/.
